# Diagnostic value of procalcitonin, C-reactive protein and lactate dehydrogenase in paediatric malignant solid tumour concurrent with infection and tumour progression

**DOI:** 10.1038/s41598-019-42264-0

**Published:** 2019-04-11

**Authors:** Fan Li, Weiling Zhang, Huimin Hu, Yi Zhang, Dongsheng Huang

**Affiliations:** Department of Pediatrics, Beijing Tongren Hospital, Capital Medical University., Beijing, (100730) China

## Abstract

Infection is a fatal complication in cancer patients that sometimes is not distinguished from tumour progression. We compared the diagnostic value of procalcitonin (PCT), C-reactive protein (CRP) and lactate dehydrogenase (LDH) in paediatric malignant solid tumour concurrent with infection and tumour progression. The 152 children enrolled were divided into infection and control groups. Each group was divided further into stable and progression groups. An intergroup comparison was made in terms of serum PCT, CRP and LDH in all children. PCT, CRP and LDH levels were significantly higher in the infection than in the control groups (*P* < 0.05). Among the controls, PCT, CRP and LDH levels were significantly higher in the progression than in the stable groups (*P* < 0.05). In diagnosing infection, the sensitivity and specificity of PCT and CRP at the cutoff values of 0.296 ng/mL and 28.13 mg/L were relatively better than those at 0.5 ng/mL and 10 mg/L, respectively. LDH had the highest correlation with tumour progression, whereas PCT had the lowest (LDH, *r* = 0.684; CRP, *r* = 0.570; PCT, *r* = 0.322). Thus, PCT has the highest value in diagnosing infection and is less susceptible to tumour progression than CRP. LDH has obvious advantages in judging tumour progression.

## Introduction

Paediatric malignant solid tumour has become an important aetiologic factor of children’s death throughout the world. Patients who receive long-term chemotherapy are prone to suffer concomitant life-threatening infections due to febrile neutropaenia, causing a rapid exacerbation in patient’s conditions and resultant increased mortality rate^1–3^. Therefore, in children, the detection and treatment of tumour concomitant with infection in the early phase of the illness help to control the patient’s conditions and further improve prognosis. Traditional infection criteria, such as bacterial and viral cultures, have disadvantages of long detection times and relatively low sensitivity^4,5^. Patients often miss the best time for infection treatment because of lack of an aetiological basis. C-reactive protein (CRP) is an acute phase protein and a sensitive biomarker that reflects the body’s inflammatory reaction^6^. It considerably shortens the time window from onset to treatment because of the convenience in sampling and short detection time, so it has been used widely for infection diagnosis. In infection patients, their CRP level often is susceptible to multiple factors, such as cancer, anti-inflammatory medication and perioperative period^7–10^, and sometimes it is difficult to judge when the detection results fluctuate greatly. Procalcitonin (PCT) is an inactive propeptide substance of calcitonin. As a systemic inflammatory reaction indicator, it is superior to other inflammatory indicators in the judgement of bacterial infection^11–13^. Existing studies have shown that its concentration is not susceptible to the impacts of immunodeficiency and use of corticosteroids^14,15^, but whether it is susceptible to tumour factors remains unclear. Lactate dehydrogenase (LDH), as a glycolytic ferment, is present in the cytoplasm of almost all histiocytes. Its level is closely associated with infectious diseases and tumour diseases, and an increased level may be related with tissue damages caused by infection or tumour cells^16–18^. Previous studies have shown that increased LDH may be interpreted as reflecting high tumour burden or tumour aggressiveness, and dynamic changes in LDH level may be useful to predict prognosis in cancer patients^19^. We detected PCT, CRP and LDH in children’s serum specimens, investigated their correlation with infection and tumour progression and further provided evidence for identification of paediatric malignant solid tumour concurrent with infection and tumour progression.

## Results

### General data

Comparison of general data (age, sex, state of tumour, tumour types and other factors) in children with malignant solid tumour showed no statistical difference between the infection and control groups (*P* > 0.05; Table 1).

**Table 1:** Comparison of general data (age, sex, state of tumour, tumour types and other factors) in children with malignant solid tumour between infection and control groups.

	Infection group	Control group	Statistic value	*P* value
Number of patients	34	118		
Age [years, *M(Q)*]	3(2.1)	3(4.0)		0.279
Gender [n (%)]				
Male	16 (47.1)	75 (63.6)	*χ*^2^ = 2.991	0.084
Female	18 (52.9)	43 (36.4)		
Tumour state [n (%)]				
Stable	20 (58.8)	74 (62.7)	*χ*^2^ = 0.169	0.681
Progression	14 (41.2)	44 (37.3)		
Tumour types [n (%)]				
Neuroblastoma	10 (29.4)	41 (34.7)	χ^2^ = 8.304	0.847
Hepatoblastoma	7 (20.6)	27 (22.9)		
Nephroblastoma	7 (20.6)	15 (12.7)		
Rhabdomyosarcoma	4 (11.8)	14 (11.9)		
PNET/Ewing’s sarcoma	2 (5.9)	6 (5.1)		
Endodermal sinus tumour	1 (2.9)	3 (2.5)		
Langerhans cell histiocytosis	1 (2.9)	3 (2.5)		
Lymphoma	0 (0)	3 (2.5)		
Osteosarcoma	1 (2.9)	1 (0.8)		
Yolk sac tumour	0 (0)	2 (1.7)		
Pulmonary blastoma	0 (0)	1 (0.8)		
Pancreatoblastoma	1 (2.9)	0 (0)		
Malignant germ cell tumour	0 (0)	1 (0.8)		
Clear cell sarcoma of the kidney	0 (0)	1 (0.8)		
Other factors [n (%)]				
Perioperative period	7 (20.6)	17 (14.4)	χ^2^ = 0.759	0.384
Non-perioperative period	27 (79.4)	101 (85.6)		

### Serum PCT, CRP and LDH levels and positive rates of PCT and CRP

Serum PCT, CRP and LDH levels and positive detection rates of PCT and CRP were significantly higher (*P* < 0.05 and *P* < 0.05, respectively) in the infection than in the control groups (Tables 2 and 3).

**Table 2:** Comparison of PCT, CRP and LDH levels between infection and control groups [*M(Q)*].

Group	Number of cases	PCT(ng/mL)	CRP(mg/L)	LDH(U/L)
Infection group	34	0.67(1.33)	97.00(88.12)	319.0(450.0)
Control group	118	0.08(0.07)	1.99(11.50)	282.5(116.0)
Statistic value	—	*Z* = −8.813	*Z* = −7.711	*Z* = −2.934
*P* value	—	0.000	0.000	0.003

**Table 3:** Number of cases and positive rates of PCT and CRP between infection and control groups [n (%)].

Group	Number of cases	PCT	CRP
Infection group	34	26(76.5%)	32(94.1%)
Control group	118	0(0%)	31(26.3%)
Statistic value	—	*χ*^2^ = 108.85	*χ*^2^ = 50.06
*P* value	—	0.000	0.000

### Serum PCT, CRP and LDH levels between the stable and progression groups among the infection and control groups

In the infection group, there was no significant difference in terms of PCT and CRP levels between the stable and progression groups (*P* > 0.05), but serum LDH levels were statistically significantly increased in the progression group (*P* < 0.05). In the control group, serum PCT, CRP and LDH levels were statistically significantly increased in the progression compared with those in the stable groups (*P* < 0.05; Table 4).

**Table 4:** Serum PCT, CRP and LDH levels between the stable and progression groups among the infection and control groups [M*(Q)*].

Group	Subgroup	Number of cases	PCT(ng/mL)	CRP(mg/L)	LDH(U/L)
Infection group	Stable group	20	0.90(1.37)	109.50(107.94)	308.0(119.0)
	Progression group	14	0.52(1.32)	57.01(92.49)	606.0(2148.0)
	Statistic value	—	*Z* = 0.840	*Z* = 1.102	*Z* = −2.170
	*P* value	—	0.416	0.274	0.030
Control group	Stable group	74	0.07(0.06)	0.94(2.16)	256.0(81.0)
	Progression group	44	0.11(0.08)	12.99(28.26)	370.0(242.0)
	Statistic value	—	*Z* = −3.487	*Z* = −6.161	*Z* = −7.397
	*P* value	—	0.000	0.000	0.000

### Value of PCT, CRP and LDH in diagnosing infection in children with malignant solid tumour

**Figure 1:**
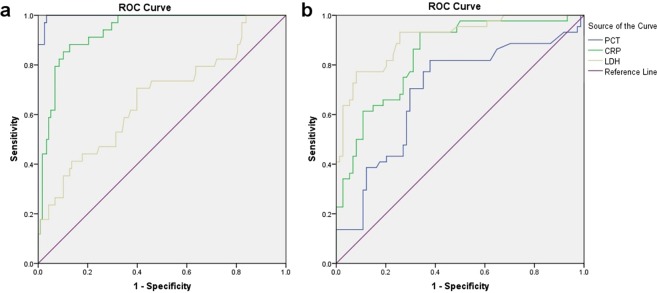
ROC curve of PCT, CRP and LDH in diagnosing infection and tumour progression in children with malignant solid tumour. (**a**) In diagnosing infection, the area under the ROC curve was 0.997 (95% CI, 0.992~1.000), 0.935 (95% CI, 0.895~0.974) and 0.665 (95% CI, 0.557~0.774) for PCT, CRP and LDH, respectively. (**b**) In diagnosing tumour progression, the area under the ROC curve was 0.692 (95% CI, 0.590~0.795), 0.840 (95% CI, 0.768~0.912) and 0.908 (95% CI, 0.853~0.963) for PCT, CRT and LDH, respectively.

The receiver operating characteristic (ROC) curve of serum PCT, CRP and LDH was drawn for all children (Fig. 1). The area under the ROC curve was 0.997 (95% confidence interval [CI], 0.992~1.000), 0.935 (95% CI, 0.895~0.974) and 0.665 (95% CI, 0.557~0.774) for PCT, CRP and LDH, respectively. Sensitivity/specificity values were 76.5%/100% and 94.1%/97.5% for PCT cutoff values of 0.5 and 0.296 ng/mL, respectively, and 94.1%/73.7% and 88.2%/87.3% when CRP cutoff values were 10 and 28.13 mg/L, respectively (Table 5).

**Table 5:** Sensitivity and specificity at different cutoff values in separate or combined diagnosis.

	Standard cutoff value	Optimal cutoff value	Cutoff at 100% specificity	Area under ROC curve
Diagnosis of infection in all children				
PCT (ng/mL)	≥0.5	≥0.296	≥0.377	0.997
Sensitivity	76.5%	94.1%	88.2%	
Specificity	100%	97.5%	100%	
CRP (mg/L)	≥10	≥28.13	≥168.18	0.935
Sensitivity	94.1%	88.2%	11.8%	
Specificity	73.7%	87.3%	100%	
LDH (U/L)		≥298.5	≥2610	0.665
Sensitivity		70.6%	11.8%	
Specificity		60.2%	100%	
PCT combined with CRP (in series)				0.996
Sensitivity	72.0%	83.0%		
Specificity	100%	99.7%		
PCT combined with CRP (in parallel)				
Sensitivity	98.6%	99.3%		
Specificity	73.7%	85.1%		
Diagnosis of progression in uninfected children				
PCT (ng/mL)		≥0.094	≥0.252	0.692
Sensitivity		70.5%	13.6%	
Specificity		70.3%	100%	
CRP (mg/L)	≥10	≥2.2	≥38.3	0.840
Sensitivity	52.3%	75.0%	22.7%	
Specificity	89.2%	73.0%	100%	
LDH (U/L)	≥295	≥300.5	≥391.5	0.908
Sensitivity	81.8%	77.3%	38.6%	
Specificity	79.7%	82.4%	100%	
LDH combined with CRP (in series)				0.944
Sensitivity	42.8%	58.0%		
Specificity	97.8%	95.2%		
LDH combined with CRP (in parallel)				
Sensitivity	91.3%	94.3%		
Specificity	71.1%	60.2%		
Diagnosis of infection combined with tumor progression in all children				
Combination of three indices (in series)				0.885
Sensitivity	57.1%	71.4%		
Specificity	91.3%	93.5%		

### Value of PCT, CRP and LDH in diagnosing tumour progression in children with malignant solid tumour

The ROC curve of serum PCT, CRP and LDH was drawn for children in the control group (Fig. 1). The area under the ROC curve was 0.692 (95% CI, 0.590~0.795), 0.840 (95% CI, 0.768~0.912) and 0.908 (95% CI, 0.853~0.963) for PCT, CRP and LDH, respectively. Sensitivity/specificity was 70.5%/70.3% for PCT cutoff values of 0.094; 52.3%/89.2% and 75.0%/73.0% for CRP cutoff values of 10 and 2.2 mg/L, respectively and 81.8%/79.7% and 77.3%/82.4% for LDH cutoff values of 295 and 300.5 U/L, respectively (Table 5).

### Value of PCT, CRP and LDH in combined diagnosis

**Figure 2:**
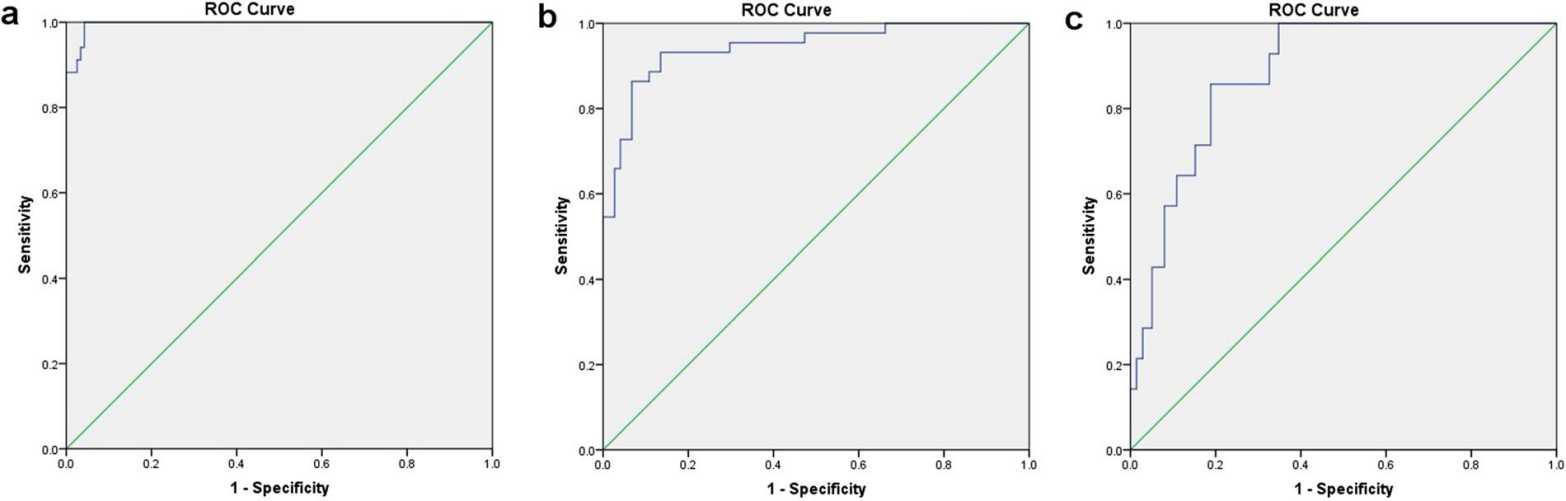
ROC curve of combined detection in diagnosing infection and tumour progression in children with malignant solid tumour. (**a**) In the diagnosis of infection by PCT combined with CRP, the area under the ROC curve was 0.996 (95% CI, 0.990~1.000). (**b**) In the diagnosis of tumour progression by LDH combined with CRP, the area under the ROC curve was 0.944 (95% CI, 0.901~0.987). (**c**) In the diagnosis of infection with tumour progression by combination of three indices, the area under the ROC curve was 0.885 (95% CI, 0.817~0.952).

The ROC curve of PCT combined with CRP in diagnosing infection, LDH combined with CRP in diagnosing tumour progression, and the combination of three indices in diagnosing infection with tumour progression were drawn, respectively (Fig. 2). The area under the ROC curve was 0.996 (95% CI, 0.990~1.000), 0.944 (95% CI, 0.901~0.987) and 0.885 (95% CI, 0.817~0.952) for PCT combined with CRP, LDH combined with CRP and the combination of three indices, respectively. Sensitivity/specificity was 72.0%/100% and 98.6%/73.7% for PCT combined with CRP in series and parallel methods (PCT ≥ 0.5 ng/mL and CRP ≥ 10 mg/L), respectively; 42.8%/97.8% and 91.3%/71.1% for LDH combined with CRP in series and parallel methods (LDH ≥ 295 U/L and CRP ≥ 10 mg/L), respectively and 57.1%/91.3% for combination of three indices in series method (PCT ≥ 0.5 ng/mL, CRP ≥ 10 mg/L, and LDH ≥ 295 U/L) (Table 5).

### Correlation analysis of serum PCT, CRP and LDH with infection and tumour progression

PCT, CRP and LDH levels were positively correlated with infection (PCT, *r* = 0.717, *P* = 0.000; CRP, *r* = 0.628, *P* = 0.000; LDH, *r* = 0.239, *P* = 0.003); PCT had the highest and LDH the lowest correlation. Serum PCT, CRP and LDH levels were positively correlated with tumour progression (PCT, *r* = 0.322, *P* = 0.000; CRP, *r* = 0.570, *P* = 0.000; LDH, *r* = 0.684, *P* = 0.000); LDH had the highest and PCT the lowest correlation.

## Discussion

Infection is the most common and one of the most fatal complications during long-term chemotherapy in children with malignant solid tumour. Early diagnosis and treatment of infection facilitate control of the patient’s conditions and improve prognosis. In cancer patients treated with myelosuppressive chemotherapy, infection often shows lack of corresponding inflammatory symptoms and signs due to the chemotherapy-induced neutropaenia^1^, with no increase in body temperature and no easy tendency to form infectious foci, such as suppuration at infection sites, so that patients often are misdiagnosed. CRP is a common clinically used infection diagnosis biomarker that is rapid and inexpensive and may be a good partner to refine the diagnosis of infection^20^. Nonetheless, neoplastic fever often is accompanied by increased CRP, so that it may not be distinguished from infection fever^21^. Early empirical antibacterial therapy often leads to fungal infection and produces antimicrobial resistance^22^. In children with malignant solid tumour accompanied by early infection, a sensitive and specific infection diagnosis method is vital to improve survival rate and prognosis. Recent studies have indicated that, as an inactive propeptide substance of calcitonin and an indicator for systemic inflammatory reaction, PCT has a high specificity and sensitivity and is superior to other inflammatory factors in the diagnosis of bacterial infection, and its concentration is not susceptible to immunodeficiency conditions and use of corticosteroids; its diagnostic value has been significantly superior to CRP and other cytokines^11–15^. In addition, recent studies also have indicated that LDH is closely associated with infectious and tumour diseases, and increased LDH level may be related to tissue damages caused by infection or tumour cells^16–18^, so that LDH detection and analysis in children with tumour progression plus infection also can provide some evidence for clinical differentiation. The combined detection of serum CRP, PCT and LDH provides a new idea for diagnosis of children with malignant solid tumour and infection and further guides the clinical treatment in a better manner.

We detected PCT, CRP and LDH in serum specimens of children and investigated their correlation with infection and tumour progression. The serum PCT, CRP and LDH levels and positive detection rates of PCT and CRP were significantly higher (*P* < 0.05 and *P* < 0.05, respectively) in the infection than in the control groups. Therefore, PCT and CRP can serve as infective indicators for diagnosis of paediatric tumour with infection. The area under the ROC curves for PCT, CRP and LDH showed that PCT was superior to CRP and that LDH did not show much value in terms of infection diagnosis. Additional studies indicated relatively low (76.5%) sensitivity and relatively good (100%) specificity at a PCT cutoff value of 0.5 ng/mL and relatively good sensitivity/specificity (94.1%/97.5%) at a cutoff value of 0.296 ng/mL. Therefore, we deduced that using 0.3 ng/mL as the threshold value for diagnosing infection in children with malignant tumour seemed reliable. The reason might be related with age, immunosuppression, tumour factors or other nonspecific factors and also with the small sample size. The sample size will be expanded, or multicentre studies will be performed for further validation.

Our study also found relatively good (94.1%) sensitivity and relatively low (73.7%) specificity at a CRP cutoff value of 10 mg/L and relatively good sensitivity/specificity (88.2%/87.3%) at a cutoff of 28.13 mg/L. Some studies have indicated that CRP level is positively correlated with tumour progression^23^. Therefore, considering the impact of tumour factors, we also deduced that using 25 to 30 mg/L as the threshold value in children with solid tumour seemed more reliable when diagnosing infection, and a prospective study should be conducted for further validation.

Regarding the relationship between these three markers and tumour progression, in the control group, PCT, CRP and LDH levels were significantly higher in the progression than in the stable groups, which indicated that the levels of the three markers were affected by tumour progression. However, in the infection group, LDH levels were significantly increased in the progression compared with those in the stable groups, and there was no significant difference in PCT and CRP levels between these groups. It may be that PCT and CRP levels were significantly affected by the infection factors and, thus, led to no difference in results. LDH levels were less affected by infection, which suggested that LDH levels in the two groups were significantly different. The results were in line with expectations. To avoid the influence of infection factors, we performed ROC curves on PCT, CRP and LDH in the control group. The area under the ROC curve was 0.693 (95% CI, 0.591~0.795), 0.837 (95% CI, 0.764~0.910) and 0.908 (95% CI, 0.853~0.963), respectively. Sometimes, when unexplained increases in CRP and LDH were found in non-infected patients, clinicians often needed to consider whether it was caused by a definite tumour progression, so this study chose the cutoff values at 100% specificity and relatively highest sensitivity as a reference. When the PCT, CRP and LDH cutoff value was greater than 0.252 ng/mL, 38.3 mg/L and 391.5 U/L, respectively, 100% of non-infected patients had tumour progression.

This study established a combined diagnosis model. PCT combined with CRP was used for the diagnosis of infection, LDH combined with CRP was used for the diagnosis of tumour progression in non-infected children, and the combination of three indices were used for the diagnosis of infection combined with tumour progression. In the combined diagnosis, the series method improved the specificity, while the parallel method improved the sensitivity. We found that the parallel method was more reliable in the combined diagnosis of infection, and the optimal cutoff was 0.296 ng/mL and 28.13 mg/L for PCT and CRP, respectively. When LDH and CRP were combined to diagnose tumour progression, the parallel method was also better, and the optimal cutoff was 295 U/L and 10 mg/L for LDH and CRP, respectively. Considering that the parallel method has no practical application value when using the combination of three indices in diagnosing infection combined with tumour progression, this study only used series method for diagnosis. Sensitivity and specificity was relatively better at the cutoff of 0.296 ng/mL, 28.13 mg/L, and 300.5 U/L for PCT, CRP and LDH, respectively. The results showed that sensitivity and specificity could be improved by selecting the appropriate method for combined diagnosis, which was helpful for the differentiation of infection from tumour progression in children with malignant tumour.

Analysis indicated that their PCT, CRP and LDH levels were positively correlated with infection (PCT, *r* = 0.717, *P* = 0.000; CRP, *r* = 0.628, *P* = 0.000; LDH, *r* = 0.239, *P* = 0.003), with PCT and CRP having a relatively high and LDH a relatively low correlation. Serum PCT, CRP and LDH levels were positively correlated with tumour progression; LDH had the highest and PCT the lowest correlation. Analysis of these results indicated that PCT and CRP had significantly higher values than LDH in diagnosing infection, and LDH and CRP also had significant advantages in judging tumour progression. Despite the positive correlation with tumour progression, PCT was less impacted by the tumour itself, so there was almost no significance when using it to judge tumour progression.

In summary, our results indicated that serum PCT and CRP are important indicators in diagnosing infection in children with malignant solid tumour. PCT is superior to CRP in diagnosing infection, and it is significantly less susceptible to tumour factors than CRP. LDH and CRP have obvious advantages in judging whether the tumour has progressed compared with PCT. The combined detection of PCT, CRP and LDH is of high diagnostic value in identifying paediatric malignant solid tumour concurrently with infection and tumour progression and is helpful in the early detection of tumour conditions. Therefore, combined detection provides the basis for the early and reasonable implementation of clinical treatment.

## Materials and Methods

### Clinical materials

This study was approved by the Ethics Committee of Beijing Tongren Hospital, Capital Medical University. All research was performed in accordance with relevant regulations, and informed consent was approved from the children and their parents. The study enrolled 152 children with malignant solid tumour admitted into the Paediatric Department of Beijing TongRen Hospital from March 2016 to March 2017. There were 51 cases of neuroblastoma, 34 of hepatoblastoma, 22 of nephroblastoma, 18 of rhabdomyosarcoma, 8 of PNET/Ewing’s sarcoma, 4 of endodermal sinus tumour, 4 of Langerhans cell histiocytosis, 3 of lymphoma, 2 of osteosarcoma, 2 of yolk sac tumour and 1 each of pulmonary blastoma, pancreatoblastoma, malignant germ cell tumour and clear cell sarcoma of the kidney (Table 1). All children were divided into infection (34 cases) and control (without infection, 118 cases) groups. Of 34 children in the infection group who had nosocomial bacterial or bacterial and viral infections, 23 had lower respiratory, 9 catheter-associated and 2 urinary tract infections. The 34 children in the infection group were subdivided into stable tumour (20) and tumour progression (14) groups. The 118 children in the control group were subdivided into stable tumour (74) and tumour progression (44) groups. All serum specimens were collected before the start of the chemotherapy cycle, and children who had taken antibiotics and corticosteroids were ruled out.

### Diagnosis criteria of tumour and infection

All malignant solid tumours were pathologically diagnosed by pathological biopsy or surgical resection. The judgement criteria for tumour progression were a 25% increase in one or more measurable lesions and appearance of new lesions^24^. The infection was diagnosed according to the definitions of the Centers for Disease Control and Prevention (CDC)^25^.

## Methods

This study was approved by the hospital’s Ethic Committee, and the informed consent was approved from the patients and their legal guardians. All experiments were performed in accordance with relevant guidelines and regulations. All children had 4 mL fasting venous blood collected before chemotherapy after admission. Serum PCT and routine biochemical tests (including CRP and LDH) were performed via the conventional detection methods at this hospital: electrochemiluminescence method to detect PCT, immunoturbidimetry to detect CRP and enzyme kinetics method to detect LDH. Infection results were judged according to PCT ≥ 0.5 (positive) and <0.5 (negative) ng/mL^26–28^ and CRP ≥ 10 (positive) and <10 (negative) mg/L^20,29^. The normal reference range of serum LDH was 110–295 U/L. The sensitivity and specificity of the combined diagnosis were evaluated by both series and parallel methods. Diagnostic rule of series method: the combined diagnosis result is considered positive when the results of all individual assays are positive. Diagnostic rule of parallel method: the combined diagnosis result is considered positive when the result of any individual assay is positive.

### Statistical analysis

The data were analysed statistically using the SPSS 22.0. Nonnormal distribution measurement data were expressed by the median (interquartile range) [*M*(*Q*)]. The nonparametric Mann–Whitney *U* test was adopted for the comparison, the χ^2^ test was adopted for the enumeration data, Spearman’s test was performed to analyse the correlation between the ranked data and the measurement data, and *P* < 0.05 indicated that the difference was statistically significant. The ROC curve of combined diagnosis was drawn according to the predicted probability after binary logistic regression analysis of PCT, CRP and LDH.

## Data Availability

The datasets used and/or analyzed during the current study are available from the corresponding author on reasonable request.

## References

[1] Crawford J, Dale DC, Lyman GH (2004). Chemotherapy-induced neutropenia: risks, consequences, and new directions for its management. Cancer..

[2] Kuderer NM, Dale DC, Crawford J, Cosler LE, Lyman GH (2006). Mortality, morbidity, and cost associated with febrile neutropenia in adult cancer patients. Cancer..

[3] López-Pousa A (2010). DELFOS Study Group. Risk assessment model for first-cycle chemotherapy-induced neutropenia in patients with solid tumours. Eur J Cancer Care (Engl)..

[4] Lagier JC (2015). Current and past strategies for bacterial culture in clinical microbiology. Clin Microbiol Rev..

[5] Leland DS, Ginocchio CC (2007). Role of Cell Culture for Virus Detection in the Age of Technology. Clin Microbiol Rev..

[6] Pepys MB, Baltz ML (1983). Acute phase proteins with special reference to C-reactive protein and related proteins (pentaxins) and serum amyloid A protein. Adv Immunol..

[7] Jukic T, Ihan A, Stubljar D (2015). Dynamics of inflammation biomarkers C-reactive protein, leukocytes, neutrophils, and CD64 on neutrophils before and after major surgical procedures to recognize potential postoperative infection. Scand J Clin Lab Invest..

[8] Shimizu T, Ishizuka M, Kubota K (2015). The preoperative serum C-reactive protein level is a useful predictor of surgical site infections in patients undergoing appendectomy. Surg Today..

[9] Bolayirli M (2007). C-reactive protein as an acute phase protein in cancer patients. Med Oncol..

[10] Nizri E (2014). C-reactive protein as a marker of complicated diverticulitis in patients on anti-inflammatory medications. Tech Coloproctol..

[11] Trimarchi H (2013). Pro-calcitonin and inflammation in chronic hemodialysis. Medicina (B Aires)..

[12] Tanrıverdi H (2015). Comparison of diagnostic values of procalcitonin, C-reactive protein and blood neutrophil/lymphocyte ratio levels in predicting bacterial infection in hospitalized patients with acute exacerbations of COPD. Wien Klin Wochenschr..

[13] R Nath S (2017). Comparative diagnostic test evaluation of serum procalcitonin and C-reactive protein in suspected bloodstream infections in children with cancer. J Med Microbiol..

[14] Rinaldi S, Adembri C, Grechi S, De Gaudio AR (2006). Low-dose hydrocortisone during severe sepsis: effects on microalbuminuria. Crit Care Med..

[15] Hatzistilianou M (2007). Serial procalcitonin responses in infection of children with secondary immunodeficiency. Clin Invest Med..

[16] Ede LC, O’Brien J, Chonmaitree T, Han Y, Patel JA (2013). Lactate dehydrogenase as a marker of nasopharyngeal inflammatory injury during viral upper respiratory infection: implications for acute otitis media. Pediatr Res..

[17] Butt AA (2002). Serum LDH level as a clue to the diagnosis of histoplasmosis. AIDS Read..

[18] Jurisic V, Radenkovic S, Konjevic G (2015). The Actual Role of LDH as Tumor Marker, Biochemical and Clinical Aspects. Adv Exp Med Biol..

[19] Zhang J (2015). Prognostic value of pretreatment serum lactate dehydrogenase level in patients with solid tumors: a systematic review and meta-analysis. Sci Rep..

[20] Clyne B, Olshaker JS (1999). The C-reactive protein. J Emerg Med..

[21] Kallio R, Bloigu A, Surcel HM, Syrjälä H (2001). C-reactive protein and erythrocyte sedimentation rate in differential diagnosis between infections and neoplastic fever in patients with solid tumours and lymphomas. Support Care Cancer..

[22] Averbuch, D. *et al*. European guidelines for empirical antibacterial therapy for febrile neutropenic patients in the era of growing resistance: summary of the 2011 4th European Conference on Infections in Leukemia. *Haematologica*. **98**, 1826–1835 (2013). Erratum in: *Haematologica*. **99**, 400 (2014). 10.3324/haematol.2013.091025 PMC3856957 24323983

[23] Verma GR (2015). Thrombocytosis and Raised CRP Levels Predicts Advanced Stage in Esophageal Carcinoma. J Gastrointest Cancer..

[24] Miller AB, Hoogstraten B, Staquet M, Winkler A (1981). Reporting results of cancer treatment. Cancer..

[25] Garner JS, Jarvis WR, Emori TG, Horan TC, Hughes JM (1988). CDC definitions for nosocomial infections, 1988. Am J Infect Control..

[26] Lai CC (2010). Diagnostic performance of procalcitonin for bacteremia in patients with bacterial infection at the emergency department. J Infect..

[27] Fazili T, Endy T, Javaid W, Maskey M (2012). Role of procalcitonin in guiding antibiotic therapy. Am J Health Syst Pharm..

[28] Quenot JP (2013). Role of biomarkers in the management of antibiotic therapy: an expert panel review II: clinical use of biomarkers for initiation or discontinuation of antibiotic therapy. Ann Int Care..

[29] Lelubre C, Anselin S, Zouaoui Boudjeltia K, Biston P, Piagnerelli M (2013). Interpretation of C-Reactive Protein Concentrations in Critically Ill Patients. Biomed Res Int..

